# From MASLD to PAD: Looking for Cardiovascular Disease Starting from Metabolic Status

**DOI:** 10.3390/medicina60111781

**Published:** 2024-10-31

**Authors:** Andrea Boccatonda, Damiano D’Ardes, Veronica Moronti, Jessica Santilli, Alessia Cipollone, Gianfranco Lessiani, Nicoletta Di Gregorio, Carla Serra, Fabio Piscaglia, Claudio Ferri, Francesco Cipollone

**Affiliations:** 1 Diagnostic and Therapeutic Interventional Ultrasound Unit, IRCCS Azienda Ospedaliero-Universitaria di Bologna, 40138 Bologna, Italy; andrea.boccatonda2@unibo.it (A.B.); carla.serra@aosp.bo.it (C.S.); 2 Department of Medicine and Aging Science, Institute of “Clinica Medica”, “G. D’Annunzio” University of Chieti-Pescara, 66100 Chieti, Italy; acipollone@live.it (A.C.); francesco.cipollone@unich.it (F.C.); 3 Department of Life, Health & Environmental Sciences and Internal Medicine, ASL Avezzano-Sulmona-L’Aquila, San Salvatore Hospital, University of L’Aquila, 67100 L’Aquila, Italyjessica.santilli@graduate.univaq.it (J.S.); nicolettadigregorio@yahoo.it (N.D.G.); claudio.ferri@univaq.it (C.F.); 4 Villa Serena Hospital, 65030 Città Sant’Angelo, Italy; gf.lessiani@gmail.com; 5 Division of Internal Medicine, Hepatobiliary and Immunoallergic Diseases, IRCCS Azienda Ospedaliero-Universitaria di Bologna, 40138 Bologna, Italy; fabio.piscaglia@unibo.it

**Keywords:** MASLD, NAFLD, PAD, atherosclerosis, vascular

## Abstract

*Background:* Peripheral artery disease (PAD) is still the least studied and evaluated form in clinical practice among atherosclerotic pathologies, despite the increased mortality and comorbidities related to it. The relationship between steatotic liver disease and an increased risk of cardiovascular disease has been extensively documented. *Methods:* The purpose of this work is to perform a review of the evidence linking NAFLD or MASLD to PAD, and examine possible clinical scenarios that arise from this new terminology. *Results:* The new definition of metabolic dysfunction-associated steatotic liver disease (MASLD) includes the presence of cardiometabolic risk factors and hepatic steatosis without any other underlying causes of hepatic steatosis; this terminology, coined in the hepatological field, could generate confusion, especially in the initial stages of its diffusion and among different medical specialists. *Conclusions:* Some recent data in the literature have strengthened the evidence of a pathological link between hepatic metabolic alteration (NAFLD or MAFLD) and PAD.

## 1. Definition of MASLD

The dangerous link between non-alcoholic fatty liver disease (NAFLD) and cardiovascular disease has been clearly established. In recent years, there has been a terminological revolution in the definition of NAFLD. The presence of hepatic steatosis and the discovery of any cardiometabolic risk factors (CMRFs) would lead to a diagnosis of metabolic dysfunction-associated steatotic liver disease (MASLD) if there are no other underlying causes of hepatic steatosis [1]. 

If additional factors contributing to steatosis are identified, this indicates a combination of causes. In cases involving alcohol, this is referred to as metabolic dysfunction and alcohol-associated steatotic liver disease (MetALD) or alcohol-associated/related liver disease (ALD), depending on the level of alcohol consumption. In the absence of obvious cardiometabolic indicators, other potential causes must be ruled out, and if none are found, it is labeled as cryptogenic steatotic liver disease (SLD), although depending on clinical assessment, it could also be considered as possible MASLD, and thus, require periodic evaluation on a case-by-case basis. In situations of advanced fibrosis/cirrhosis, steatosis might not be present, necessitating clinical judgment based on CMRFs and the absence of other causes.

Moreover, it is important to underline the potential classification of steatotic liver disease (SLD). The most common causes of SLD include metabolic dysfunction-associated steatotic liver disease (MASLD), characterized by hepatic steatosis, alongside at least one cardiometabolic risk factor (CMRF) and, in the absence of other identifiable causes, alcohol-associated/related liver disease (ALD), and a combination of the two (MetALD). Individuals with MASLD and concurrent steatohepatitis are classified as having metabolic dysfunction-associated steatohepatitis (MASH). Within the MetALD category, there exists a spectrum where the relative contributions of MASLD and ALD vary. To reflect the current research, specific limits have been established for weekly and daily alcohol consumption, recognizing that the effects of alcohol intake can differ among individuals. Other potential causes of SLD are considered separately due to their distinct pathophysiology, as is common in clinical practice. It is important to note that multiple causes of steatosis can coexist. In cases where there is uncertainty and the clinician suspects metabolic dysfunction despite the absence of CMRF, further testing, such as HOMA-IR and oral glucose tolerance tests, may be warranted to assess for early MASLD. Those with no identifiable cause (cryptogenic SLD) may potentially be reclassified in the future pending advancements in our understanding of disease pathophysiology. Furthermore, the ability to diagnose MASLD affirmatively allows for the coexistence of other liver diseases with MASLD, such as MASLD alongside autoimmune hepatitis or viral hepatitis. Regarding MASLD and increased alcohol consumption, the weekly intake is between 140 and 350 g for females and between 210 and 420 g for males (daily average between 20 and 50 g for females and between 30 and 60 g for males).

In the subclass of monogenic diseases associated with SLD, examples include lysosomal acid lipase deficiency (LALD), Wilson disease, hypobetalipoproteinemia, and inborn errors of metabolism. The miscellaneous class of SLD encompasses etiologies such as HCV, malnutrition, celiac disease, and human immunodeficiency virus (HIV).

Within the atherosclerotic pathology, PAD continues to be the least studied and evaluated form in clinical practice, despite the increased mortality and comorbidities related to it. The new definition of MASLD, coined in the hepatological field, can generate confusion, especially in the initial stages of its diffusion and among different medical specialists. Therefore, the purpose of this work is to perform a review of the evidence linking NAFLD or MASLD to PAD, and examine possible clinical scenarios that arise from this new terminology.

## 2. MASLD and Cardiovascular Diseases

The relationship between SLD and cardiovascular events (CVEs) has been well documented. MASLD has been shown to induce a higher risk of CVE by 45% (95% CI: 1.31–1.61) in a recent systematic review involving 5,802,226 individuals and 99,668 patients with fatal and non-fatal CVE by performing a 6.5-year median follow-up [2]. CVE risk is directly related to the severity of SLD, particularly in the phase of fibrosis (HR: 2.50; 95% CI: 1.68–3.72) [2].

Koulaouzidis et al. evaluated four observational works involving 10,060 patients, showing that SLD was significantly associated with the worsening of coronary artery calcification (CAC) (OR: 1.5; 95% CI: 1.34–1.68) [3].

Another study involving >2.5 million patients across twenty studies revealed that SLD was correlated with ischemic stroke (OR: 1.41; 95% CI: 1.29–1.55), myocardial infarction (OR: 1.66; 95% CI: 1.39–1.99), heart failure (OR: 1.62; 95% CI: 1.43–1.84), and atrial fibrillation (OR: 1.27; 95% CI: 1.18–1.37) [4].

In a recent Korean nationwide work involving 9,584,399 individuals, participants were analyzed according to four characteristics: NAFLD only, metabolic-associated fatty liver diseases (MAFLD) only, both conditions, or neither fatty liver disease [5]. The primary CV outcome was a composite of myocardial infarction, ischemic stroke, heart failure, or CV death [5]. Over a median follow-up period of 10.1 years, 8.9 million individuals without CV events were tracked [5]. Both NAFLD and MAFLD were related to an increased CVE risk [5].

By establishing the neither-FLD subjects as a reference, the NAFLD group showed an HR of 1.09 (95% CI: 1.03–1.15) for CVE; the patients with MAFLD displayed HRs of 1.43 (95% CI: 1.41–1.45) and 1.56 (95% CI: 1.54–1.58) in the subjects with both fatty liver diseases [5]. Therefore, employing MAFLD, with the current change in nomenclature, may help to better identify patients with fatty liver disease and a high risk of CVE.

A meta-analysis revealed that individuals with MASLD face a significantly higher risk of several health conditions compared to those without MASLD. These include increased likelihood of CVE (HR: 1.49, CI: 1.34–1.64), stroke (HR: 1.55, CI: 1.37–1.73), carotid atherosclerosis (OR: 1.18, CI: 1.00–1.38), PAD (OR: 1.32, CI: 1.05–1.68), cardiovascular mortality (HR: 1.28, CI: 1.03–1.53), total mortality (HR: 1.24, CI: 1.13–1.34), cancer-related mortality (HR: 1.27, CI: 1.01–1.54), liver-related mortality (HR: 2.76, CI: 1.07–7.13), CKD (HR: 1.53, CI: 1.38–1.68), HF (HR: 1.67, CI: 1.58–1.76), and obstructive sleep apnea (OR: 6.80, CI: 1.81–25.6) [6]. A recent meta-analysis, which included 15 studies and more than 10 million individuals, found that those with NAFLD had an increased risk of both all-cause mortality (HR: 1.32, 95% CI: 1.09–1.59) and CV mortality (HR: 1.22, 95% CI: 1.06–1.41) [7].

A more recent meta-analysis, which included 10,592,851 participants (2.9% diagnosed with NAFLD) and an average follow-up of 10 ± 6 years, found no significant association between NAFLD and increased risk of all-cause mortality (OR: 1.14; 95% CI, 0.78–1.67) or cardiovascular mortality (OR: 1.13; 95% CI, 0.57–2.23) [8]. NAFLD was associated with a 60% higher risk of both myocardial infarction and stroke (OR: 1.6; 95% CI, 1.5–1.7 and OR: 1.6; 95% CI, 1.2–2.1, respectively), a 70% increased risk of atrial fibrillation (OR: 1.7; 95% CI, 1.2–2.3), and a 2.3-fold higher risk of major adverse CVE and cerebrovascular events (OR: 2.3; 95% CI, 1.3–4.2) [8]. A systematic review and meta-analysis of 4725 individuals with NAFLD examined the relationship between liver fibrosis and subclinical atherosclerosis. Subclinical atherosclerosis was evaluated through markers such as arterial stiffness, CAC score, and intima-media thickness [9]. The analysis found that fibrosis degree was related to subclinical atherosclerosis (OR: 2.18; CI, 1.62–2.93) [9]. The OR values were 1.64 (95% CI, 1.22–2.20) for fibrosis stage ≥F1, 2.22 (95% CI, 1.37–3.62) for stage ≥F2, and 3.42 (95% CI, 1.81–6.46) for stage ≥F3 [9].

Table 1 summarizes MASLD and CV diseases.

## 3. Pathophysiology of Increased Cardiovascular Risk in MASLD Patients

**Figure 1:**
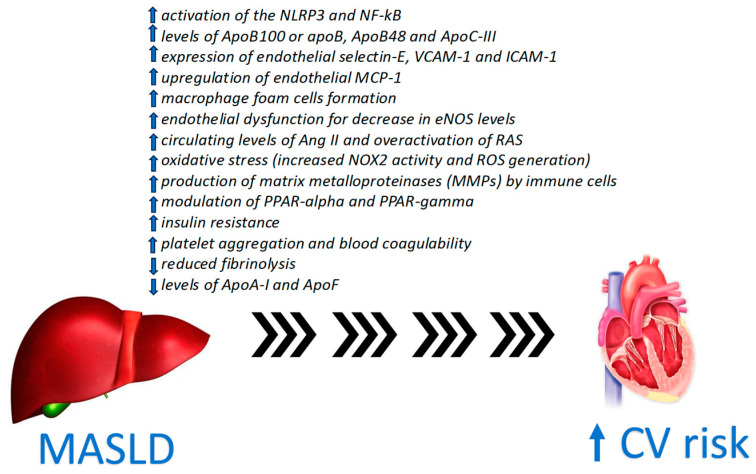
Summarizes pathophysiology of increased CV risk in MASLD.

Considerable epidemiological evidence connects MASLD with atherosclerotic cardiovascular disease [10]. CV disease stands as the primary cause of mortality among individuals with MASLD [11]. Numerous molecular processes, as well as cellular and metabolic factors, that contribute to atherosclerosis plaque development and rupture in MASLD have been elucidated [12,13,14,15] (Figure 1).

Around 60–70% of MASLD patients present with dyslipidemia [16]. Atherogenic dyslipidemia is characterized by elevated plasma triglyceride levels featuring higher triglyceride-rich lipoproteins like very-low-density lipoproteins (VLDL) and intermediate-density lipoproteins (IDL). Moreover, there is a prevalence of small and dense low-density lipoprotein particles and a diminished level of high-density lipoprotein (HDL) cholesterol [17]. Abnormalities in the levels of apolipoproteins commonly observed in MASLD patients include increased levels of ApoB100 or apoB, ApoB48 and ApoC-III [17]. ApoC-III might enhance the secretion of chylomicrons and VLDL, may inhibit lipoprotein lipase and hepatic lipase activities, and may disrupt apoE binding to liver receptors. This interference results in the reduced uptake of triglyceride-rich lipoprotein remnants [18,19].

Decreased levels of ApoA-I and ApoF were demonstrated in MASLD patients [20]. Recent research indicates a reduction in apoF levels in individuals with hepatic steatosis, with decreased hepatic apoF expression correlating with an atherogenic lipid profile characterized by elevated levels of triglycerides, non-HDL cholesterol, and diminished levels of HDL-cholesterol [21]. The low expression of apoF leads to a decrease in hepatic lipoprotein remnant clearance [21].

MASLD can induce a higher expression of endothelial selectin-E, VCAM-1 (vascular cell adhesion molecule-1), and ICAM-1 (intercellular cell adhesion molecule-1) [13,14]. These molecules facilitate the adhesion of circulating monocytes to the endothelial surface, thus initiating a cascade of events that ultimately results in atherosclerotic plaque formation. Additionally, there is an upregulation of endothelial MCP-1 (monocyte chemoattractant protein-1), which enhances the chemoattraction and trans-endothelial migration of adhered monocytes into the subendothelial space [13]. When monocytes migrate into the arterial intima, they differentiate into macrophages. These macrophages take up cholesterol esters from oxidized LDL and triglyceride-rich lipoprotein remnants via surface scavenger receptors. As they accumulate cholesterol esters, the macrophages become foam cells, which build up in the arterial intima, creating a fatty streak, the earliest detectable lesion in atherosclerosis.

In MASLD, there is an elevated expression of macrophage scavenger receptors in the arterial intima. This includes receptors like SR-A (scavenger receptor class A), CD36, LOX-1 (lectin-like oxidized low-density lipoprotein receptor-1), and the apoB48 receptor (apoB48R) [13].

Moreover, the expression of ATP-binding cassette transporter A1 (ABCA1) on macrophage surfaces is lower in MASLD, possibly due to increased amounts of fetuin-A [13]. ABCA1 facilitates the release of free cholesterol from arterial intima-resident macrophages to apoA-1, forming pre-β HDL or nascent HDL [22]. Consequently, decreased ABCA1 expression on macrophage surfaces promotes foam cell formation. Patients with MASLD are characterized by higher amounts of M1 macrophages releasing inflammatory molecules [14].

In MASLD, low-grade inflammation in the vasculature is exacerbated by the increased activation of the NLRP3 (NOD-like receptor protein 3) inflammasome and NF-kB (nuclear factor-kappa B). This heightened activation leads to elevated levels of inflammatory cytokines and factors, such as IL-1β, IL-6, TNF-α, C-reactive protein, and fetuin-A [13].

Fetuin-A represents a circulating glycoprotein synthesized by the liver and adipose tissue, and it plays a significant role by stimulating the expression of IL-6, selectin-E, ICAM-1, and MCP-1 in endothelial cells [23]. Moreover, it can enhance the formation of macrophage foam cells by inducing scavenger receptors CD36 and SR-A synthesis, as well as acyl-CoA:cholesterol acyltransferase-1 (ACAT-1); it is involved in converting free cholesterol into cholesterol esters within macrophages. Additionally, fetuin-A downregulates ABCA1, further promoting foam cell formation, and favoring the generation of vascular smooth muscle cells.

Endothelial dysfunction is closely associated with MASLD. In MASLD, there is a decrease in endothelial nitric oxide synthase (eNOS) levels, leading to reduced synthesis of NO by the endothelium—an endogenous molecule known for its anti-atherogenic properties [13,14]. Furthermore, MASLD is marked by the elevated endothelial expression of asymmetric dimethyl arginine (ADMA) and endothelin-1 (ET-1). ADMA inhibits eNOS (endothelial nitric oxide synthase), whereas ET-1 induces vasoconstriction, increases vascular smooth muscle cell tone, promotes their proliferation, and contributes to thrombosis, inflammation, and eNOS uncoupling [13,14,24]. When eNOS is not coupled, it produces the highly reactive superoxide anion rather than nitric oxide (NO). ET-1 induces the production and activation of arginase-2 in endothelial cells and macrophages, which in turn enhances the production of ROS [25]. At the endothelial level, arginase competes with eNOS for the L-arginine, thereby decreasing the availability of L-arginine for NO production and, as a result, reducing the bioavailability of NO [25].

Moreover, oxidative stress contributes to endothelial cell dysfunction. Individuals with NAFLD exhibit elevated NOX2 activity, a specific isoform of nicotinamide adenine dinucleotide phosphate (NADPH) oxidase responsible for generating reactive oxygen species (ROS) [26]. NADPH oxidase stands as the primary source of ROS in humans. When the ROS anion superoxide interacts with nitric oxide (NO), the latter is deactivated, resulting in endothelial dysfunction [27]. ROSs play a pivotal role in oxidizing LDL and transforming macrophages into foam cells [28]. Additionally, elevated homocysteine levels, observed in MASLD, further contribute to endothelial dysfunction by impairing NO formation [28]. In NAFLD, increased levels of IL-1β, IL-6, and CRP contribute to systemic inflammation, thereby worsening endothelial dysfunction [28].

In MASLD, the regulation of VSMC mitogenesis is disrupted, thus resulting in heightened proliferation, hypertrophy, and migration of these cells [13]. The migration of VSMC from the tunica media (the middle layer of the arterial wall) to the intima leads to the development of neointima within the atherosclerotic plaque.

Subjects with MASLD exhibit elevated circulating levels of angiotensin II (Ang II) and an overactive intrahepatic renin–angiotensin system (RAS). This imbalance plays a significant role in advancing both liver and cardiovascular complications linked to MASLD [14].

Activated human hepatic stellate cells express essential components of the RAS and generate Ang II. This production plays a crucial role in promoting liver fibrosis and driving other pathological processes within the liver. The activation of the RAS, along with increased Ang II levels, causes vasoconstriction and enhances oxidative stress. Additionally, it upregulates the expression of endothelial markers such as ICAM-1, VCAM-1, selectin-P, and MCP-1, all of which contribute to the development and progression of atherosclerosis [14]. Furthermore, increased activity of the sympathetic nervous system triggers vasoconstriction and raises the risk of CV disease, thereby amplifying vulnerability to CV complications [14].

In MASLD, immune cells exhibit the increased production of matrix metalloproteinases (MMPs) 2, 9, and 12. These enzymes play a significant role in tissue remodeling and inflammation, contributing to the progression of liver damage in MASLD [13]. These MMPs degrade the extracellular matrix proteins within the fibrous cap of atherosclerotic plaques, thus weakening the structure and making the plaques more vulnerable or unstable. This increased vulnerability heightens the risk of plaque erosion or rupture, potentially triggering acute coronary syndromes, such as heart attacks or other severe cardiovascular events [29].

Moreover, it is interesting to underline a possible role of peroxisome proliferator-activated receptors (PPARs) in the pathophysiology of MASLD and cardiometabolic-related diseases. Indeed, PPAR-alpha is primarily involved in the regulation of genes associated with fatty acid oxidation and in lipid metabolism; PPAR-alpha promotes the synthesis of enzymes involved in lipid metabolism by activating target genes, thus helping to lower triglyceride levels and improve liver function. PPAR-alpha has an anti-inflammatory activity involved in reducing fibrosis in the liver [30]. PPAR-gamma enhances insulin sensitivity, and is involved in adipose tissue differentiation, playing a role in storing excess lipids in adipocytes, influencing systemic lipid levels and, consequently, liver health [30].

### 3.1. MASLD and Insulin Resistance

MASLD is marked by insulin resistance, a core pathological factor that significantly contributes to the onset of atherogenic dyslipidemia, endothelial dysfunction, low-grade vascular inflammation, and oxidative stress. These mechanisms are fundamental in the development of atherosclerotic disease. Upon insulin binding to its receptors, it activates distinct signaling pathways within endothelial cells, which further drive these pathological processes:

(a) The phosphatidylinositol 3-kinase (PI3-K) pathway controls eNOS activity, resulting in the production of NO, a potent vasodilator with protective properties against atherosclerosis;
(b) The mitogen-activated protein kinase (MAPK) pathway promotes cell proliferation and upregulates the expression of VCAM-1 and ICAM-1. It also facilitates the synthesis and release of endothelin-1 (ET-1), which plays a significant role in vascular functions [31].

In insulin resistance conditions, the PI3-K pathway shows reduced sensitivity to insulin, while the MAPK pathway remains responsive. This selective disruption in insulin signaling results in abnormal cellular responses, fostering processes that promote the development of atherosclerosis [31]. Consequently, insulin resistance reduces endothelial NO production, while simultaneously increasing the expressions of VCAM-1, ICAM-1, and ET-1. This process also triggers VSMC proliferation and elevates oxidative stress, all of which play key roles in promoting vascular dysfunction and the development of atherosclerosis [31].

### 3.2. MASLD and Platelet Aggregation, Coagulation, and Fibrinolysis

In MASLD, there is an increase in platelet aggregation and blood coagulability, along with a decrease in fibrinolysis [1]. This is characterized by elevated levels of fibrinogen, multiple coagulation factors (VII, VIII, IX, XI, XII, and von Willebrand factor), soluble CD40 ligand, and plasminogen activator inhibitor-1 (PAI-1). Simultaneously, there is a reduction in tissue-type plasminogen activator (tPA) and key endogenous anticoagulants like antithrombin III and protein C. Therefore, MASLD is related, and promotes a prothrombotic and anti-fibrinolytic environment [15].

## 4. PAD: Clinical Overview

The terms “peripheral arterial disease” and “peripheral vascular disease” are frequently used freely [32]. These terms have often included different atherosclerotic diseases as compared to cardiac diseases such as atherosclerotic disease in the carotid artery, renal artery, leg artery and aorta. In this review, we will refer to peripheral arterial disease (PAD), according to AHA, as atherosclerotic plaque from the aortoiliac segments downwards [33].

PAD is a risk factor for cardiovascular events and all-cause mortality [34,35,36]; therefore, it is important to diagnose and prevent it as soon as possible [37] (Table 2).

### 4.1. Symptoms

Intermittent claudication is the main symptom of PAD, and it is defined as a calf pain induced by stress that begins with walking, does not happen at rest, and regresses within 10 min of stopping the effort. About 70 to 90% of people affected by PAD report no intermittent claudication (asymptomatic) and others report atypical symptoms, i.e., pain not involving the calf or regressing with walking [38,39]. Given the high prevalence of asymptomatic or atypical symptoms of PAD, clinicians should investigate walking ability and leg symptoms in all patients older than 50 years [40]. People should be educated about symptoms that may indicate PAD, such as declining walking ability, weakness and leg discomfort [40]. Improving PAD awareness is an important goal for Healthy People 2030 [40].

### 4.2. Risk Factors and Screening

Cardiovascular diseases, cerebrovascular diseases, diabetes and smoking habits are risk factors for PAD, thus individuals with these diseases should undergo ABI testing [40].

Hypertension, dyslipidemia and sedentary lifestyle are also considered risk factors for the development of PAD [41]. Smokers and individuals affected by diabetes have double the risk of PAD compared with those without T2DM and non-smokers [42,43]. The risk for PAD is equal between ex-smokers and non-smokers after 30 years of smoking cessation, whilst the risk of cardiovascular diseases takes 20 years [44].

Dyslipidemia increases the risk of developing PAD, and levels of apolipoprotein B and lipoprotein (A) have been demonstrated as independent risk factors for cardiovascular disease [45,46,47]. Small low-density lipoprotein is associated with PAD, better than low-density lipoprotein cholesterol; this association was reported in a recent analysis from the Women’s Health Study [48].

The ARIC study (Atherosclerosis Risk in Communities) demonstrated that systolic blood pressure has a graded association with PAD [49]. Instead, diastolic blood pressure is associated with a significantly elevated risk of PAD only above 90 mmHg [49].

Inflammatory markers, i.e., IL-6 (interleukin-6) and CRP (C reactive protein), are elevated in patients affected by symptomatic PAD, data confirmed in the Edinburgh Artery Study [46].

Patients with MAFLD and/or metabolic syndrome have an underlying pro-inflammatory milieu. Previous studies on NAFLD patients have demonstrated the presence of an increased risk of atherosclerosis [50]. The risk of atherosclerosis in those patients was also associated with advanced fibrosis (29% increased risk of atherosclerosis) [51]. The risk of fibrosis deterioration was independently associated with an ABI diagnostic for PAD, especially in patients with insulin resistance [52].

Therefore, PAD and insulin resistance are synergic in fibrosis progression, and insulin resistance is one of the main risk factors in the onset of PAD as well as fibrosis progression [52]. Inflammatory settings and oxidative stress are common risk factors in PAD and NAFLD, thus suggesting their relationship [53]. A recent prospective analysis demonstrated the association between MAFLD and subclinical atherosclerosis, i.e., elevated carotid intima-media thickness and elevated arterial stiffness [54].

Zou et al. carried out a cross-sectional study on patients with T2DM [55]. Patients with NAFLD had an increased risk of developing PAD compared with patients without NAFLD [55]. The association between NAFLD and PAD remained significant when considering age, gender, smoking habits, diabetes treatment, BMI, systolic blood pressure, triglyceride and total cholesterol [55]. 

In this context, we can consider MAFLD as a major metabolic disease, and not just liver disease, with an increased risk of cardiovascular diseases [53,56,57].

Song et al., in a prospective study of the Chinese Population, explored the association between MAFLD and the risk of PAD, diagnosed by ABI < 0.9 or ≥1.3 [37]. This study confirmed that PAD was higher in patients affected by MAFLD than those without, and that the incidence of PAD was directly associated with the number of metabolic comorbidities [37]. It is important to underline that this study demonstrated that MAFLD was an independent risk factor of both the onset and progression of PAD [37].

Cardiovascular risk is equally high in symptomatic and asymptomatic PAD patients [58]. Therefore, it is important to take an ABI measurement in all patients with high cardiovascular risk, regardless of the symptoms, such as those with MAFLD, metabolic syndrome and diabetes [59].

AHA recommends ABI testing in adults over 65 years old or over 50 years old with traditional risk factors, for the screening of PAD [59,60]. The results of a recent Danish trial [61] underline that to reduce mortality, it is important to perform a comprehensive screening of PAD, hypertension and aneurysm of the abdominal aorta, in people from 65 to 74 years old, followed by the best medical treatment (statin, aspirin, anti-hypertensive therapy) and surgical aneurysm repair, if needed.

Not only cardiovascular risk factors are related to PAD. Low education levels, low family income and isolation increase the risk of PAD regardless of cardiovascular risk factors [33].

Therefore, these patients should also be screened for PAD.

### 4.3. ABI/TBI

AHA defined PAD as an obstruction due to atherosclerotic plaque from the aortoiliac segments downwards [33]. People with intermittent claudicatio should be tested with ABI [40]; it is a non-invasive diagnostic test, which is simple to perform, inexpensive, and accurate for diagnosis of PAD [62].

It consists of a Doppler-measurement of systolic pressure in the brachial, posterior tibial and dorsal pedis arteries in both arms and legs; then, the highest or the mean of posterior tibial and dorsal pedis artery pressures is divided by the highest or the mean of two measures of homolateral brachial artery pressure, for each hemisoma [38].

An ankle–brachial index ≤0.90 is considered diagnostic for PAD (arterial stenosis ≥ 50%) [62]. An ABI from 0.9 to 1.09 can indicate mild atherosclerosis of the lower extremity, but it is associated with higher cardiovascular events than in people with ABI from 1.1 to 1.3 [63,64]

An ABI ≤ 0.90 is 57% sensitive and 83% specific for arterial stenosis ≥ 50%, compared to angiography, which is 79% sensitive and 99% specific [38]. The ABI sensitivity can be improved by re-testing after stress exercise, i.e., treadmill test or heel raise test [38]. Several factors influence ABI sensitivity and specificity, such as the presence of calcium in the arterial wall, arterial depth and the presence of multiple occlusions [65].

An ABI > 1.40 is due to medial artery calcification that increases arterial stiffness; this condition is common in patients with diabetes or chronic kidney disease. The increased stiffness results in high systolic pressure at the ankle, hence ABI is abnormally elevated even in patients with PAD [38]. In these cases, it is necessary to measure toe–brachial index (TBI), because digital arteries are rarely affected by medial calcification [59,66,67].

TBI is the ratio between toe systolic pressure and brachial systolic pressure; when it is ≤0.70, it is diagnostic for PAD [59].

“Take the socks off” is mandatory for clinicians to facilitate the diagnosis of PAD in high-risk patients [40].

Ultrasound also plays a role after endovascular or surgical treatment, assessing the effectiveness of the treatment.

### 4.4. Other Imaging

Computer tomography angiography and magnetic resonance angiography are useful for the indication of surgical or endovascular treatment [68]. Catheter-based angiography is the gold standard for evaluating the presence of PAD, but it is considered in patient candidates for endovascular revascularization [59].

## 5. Common Clinical Evidence Between MASLD and PAD

A recent study conducted by Song et al. involved 102,115 participants who underwent routine health checkups. PAD was evaluated using the ABI, while MAFLD was diagnosed via abdominal ultrasound [37]. The study revealed that patients with MAFLD had a greater prevalence of PAD compared to those without MAFLD, with a prevalence rate of 2.7% in the MAFLD group versus 2.2% in the non-MAFLD group [37]. The adjusted odds ratio for this increased risk was 1.30 (95% CI: 1.19–1.42, *p* < 0.001), demonstrating a statistically significant association with MAFLD [37].

In a prospective cohort study of 6833 participants, followed for an average of 2.76 years (standard deviation: 1.36 years), baseline MAFLD was linked to a higher risk of developing PAD. The adjusted HR for this association was 1.67 (95% CI: 1.17–2.38, *p* = 0.005), signifying a statistically significant increase in risk [37].

Additionally, the risk of PAD increased with the accumulation of metabolic abnormalities in individuals with MAFLD [37]. The risk of PAD was particularly pronounced in participants with MAFLD who did not have other metabolic comorbidities [37].

In another study involving 889 subjects with diabetic foot ulcers (DFUs), the participants were divided into two groups: non-MAFLD (*n* = 643) and MAFLD (*n* = 246). They were followed up every 6 months over a period of 10.9 years, with a median follow-up time of 63 months [69]. Out of the 889 subjects, 214 (24.07%) experienced major adverse cardiovascular and cerebrovascular events (MACCEs). MAFLD was found to be independently associated with a higher risk of MACCEs (*p* < 0.001), including specific events such as non-fatal myocardial infarction (*p* = 0.04), non-fatal stroke (*p* = 0.047), coronary artery revascularization (*p* = 0.002), HF (*p* = 0.029), and all-cause mortality (*p* = 0.021) [69]. Compared to the non-MAFLD group (HR = 1), individuals with diabetic foot ulcers (DFUs) and MAFLD had a 2.64-fold-higher risk of developing MACCEs (*p* < 0.001; *p* for interaction = 0.001) within the PAD subgroup. Furthermore, the MAFLD group demonstrated a significantly higher cumulative incidence of MACCEs, as indicated by log-rank tests (all *p* < 0.05) [69].

All reported evidence is summarized in Table 3.

## 6. Therapeutical Options in Patients with MASLD and PAD

Lifestyle changes play a fundamental role in the managing of PAD and in the reduction of the risk of MASLD, by helping people to quit smoking and stop drinking [10]; patients with PAD who continue smoking have grater rates of adverse lower extremity outcomes [71]. It is necessary to promote smoking cessation by helping patients both with pharmacotherapy and smoking cessation programs, as indicated in the AHA/ACC guidelines [59]. A low-calorie diet, possibly a Mediterranean diet rich in fruit and vegetables, can reduce intrahepatic fat content; moreover, ketogenic diet also exerts a positive effect in reducing body weight and insulin resistance in patients with MAFLD [72]. 

Physical exercise represents the first-line therapy to improve walking impairment in people with PAD [59]. Supervised treadmill exercise has a clear effect on improving walking distances, and facilitates greater comfort and familiarity in those who approach this activity [73]. As reported in several randomized trials, arm and leg ergometry exercise improved cardiovascular fitness beyond that of walking distance [59,70,74]. Home-based exercise, defined as exercise carried out in or around the home outside of a medical center, can significantly improve 6-min walk distance, avoid the inconvenience and costs of supervised exercise, and, when conducted at high intensity, can induce ischemic leg symptoms significantly [62,75]. Physical exercise, both aerobic and resistance training, also represents an important therapeutic intervention in the management of MASLD [76,77].

AHA/ACC guidelines recommend aspirin or clopidogrel for patients with symptomatic PAD; iclopidogrel can reducecardiovascular events significantly more than aspirin, but in some randomized trials, the same medication plus aspirin did not reduce cardiovascular events more than aspirin alone in patients with PAD [78]. In the PEGASUS-TIMI trial, patients treated with ticagrelor 90 mg showed greater reductions in acute limb ischemia and lower extremity events compared to clopidogrel [79]. The medical treatment that has shown the best results to date in the management of PAD is rivaroxaban 2.5 mg twice daily combined with 100 mg aspirin, which reduced the combined primary outcomes of cardiovascular mortality, stroke, myocardial infarction and major adverse lower extremity events (COMPASS trial) [80]. Cilostazol promotes vasodilation, in particularly in femoral arteries, exerts antithrombotic activities, and was associated with a 43 m improvement in treadmill walking distance compared with placebo; however, this drug cannot be prescribed for patients with PAD who have heart failure of any severity [81].

The usefulness of the aspirin was also found in patients with NAFLD, reducing liver fibrosis indexes [82], and it is recommended in the presence of established atherosclerotic disease [10].

Patients with NAFLD often have other comorbidities, like hypertension, T2DM and kidney disease, and an increased expression of components of the RAS was demonstrated in animals developing liver steatosis; these data reinforce the theory about the possible mechanisms involved in the pathology and experimental data suggesting that the use of RAS antagonists can reduce NAFLD progression in hypertensive patients [83]. On the other hand, blood pressure control is relevant even in patients with PAD, and the guidelines recommend a treatment goal of less than 130/80 mmHg. The best medications used to achieve this are angiotensin-converting enzyme inhibitors and angiotensin receptor blockers [59].

Several studies have demonstrated the usefulness and a possible protection of statins in patients with MASLD; this is characterized by an increase in hepatic free cholesterol with much higher values depending on the severity of liver damage [84,85]. It seems that the beneficial effect of the statin occurs above all in patients negative for the PNPLA3 mutation [86]. Statins also play a crucial role in the management of PAD, as shown in the Heart Protection Study, where simvastatin 40 mg significantly reduced the combined outcome of fatal and nonfatal coronary events, stroke, and revascularization compared with placebo [87]. The AHA/ACC guidelines recommend high-dose statins to attain an LDL reduction of at least 50%, and recommend ezetimibe for patients with PAD whose LDL level is not in range [88]. In the Fourier trial, evolocumab compared with placebo reduced cardiovascular events in patients with PAD when the LDL level remained outside of the target range, despite statin and ezetimibe therapy [89]. The same medication could be used in patients with NAFLD who cannot obtain good values of LDL, despite the use of other hypolipidemic drugs [10].

Omega-3 fatty acids can decrease lipogenesis, and some evidence suggests that they may decrease liver enzymes, but the real effect on liver damage is still uncertain [90,91].

In some randomized controlled trials with patients affected by NAFLD, metformin was associated with protection from hepatocellular carcinoma development and with lower mortality in patients with cirrhosis [92]; therefore, it would be appropriate not to stop metformin in NAFLD patients with advanced liver disease unless contraindicated by severe hepatic failure [93].

Pioglitazone, another insulin sensitizer, can decrease hepatic fat accumulation and leads to improved liver damage, although the long-term impact on inflammation and fibrosis is doubtful [94].

Liraglutide, a GLP-1 agonist, increased the rate of NASH resolution, reduced liver fibrosis progression and is associated with lower rates of diabetic foot ulcer-related amputations; those benefits are mostly dependent on weight loss, and it seems had no effect on foot infections or peripheral revascularization rates [95,96].

SGLT2 inhibitors have potential beneficial effects on NAFLD. In a recent trial, empaglifozin demonstrated a histological improvement of hepatic steatosis and fibrosis in patients with NASH and T2DM [97]; furthermore, some preliminary evidence suggests that patients with PAD may even benefit from this drug, although two trials on canaglifozin suggested excess amputation rates [98].

Some studies hve evaluated the impacts of two nuclear receptor agonists, obeticholic acid and elafibranor, on histological damage, liver-related outcomes and survival in NASH patients, acting against hepatic fat accumulation, inflammation and fibrosis; elafibranomer has shown beneficial effects on several metabolic syndrome traits. (NCT02704403) [99]. Other drugs that appear to be promising in the managing of MAFLD are fibroblast growth factors (FGFs) 19 and 21 agonists and PPAR agonists, which act mainly on the metabolism of fats and glucose [100].

In patients with PAD, revascularization is applied only when guideline-recommended medical therapies, including supervised exercise, fail [60], while bariatric surgery in obese patients with NAFLD may be a possible strategy to drastically reduce the body weight and to improve steatohepatitis histologically [15,100].

Resmetirom is a new promising agent against MASLD; it is an oral drug, which is liver-directed, and is a thyroid hormone receptor beta (THR-β)-selective agonist in development for the treatment of hepatic steatosis with liver fibrosis. This drug was evaluated in a phase-3, randomized, controlled trial on 966 patients showing good efficacy and tolerance [101]. Fibrosis improvement by at least one stage with no worsening of the hepatic steatosis activity score was achieved in 24.2% of the patients in the 80 mg resmetirom group and 25.9% of those in the 100 mg resmetirom group compared with 14.2% of those in the placebo group (*p* < 0.001 for both comparisons with placebo) [101].

In any case, given the complex cardiometabolic factors that characterize MASLD and PAD, the therapeutic strategy is always a combination of multiple therapies that act on different fronts to modify the course of both pathologies and interrupt the synergy that these two diseases can generate when they are associated.

## 7. Conclusions

**Figure 2:**
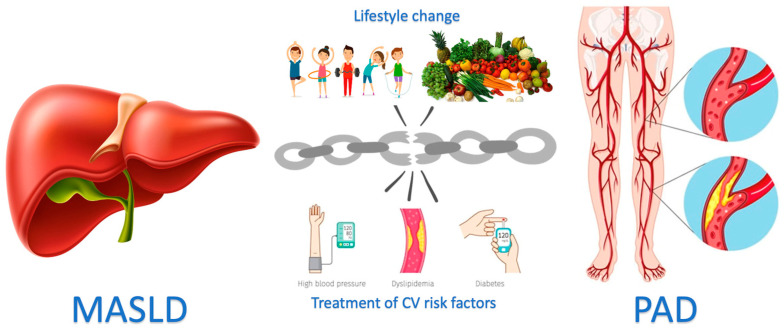
Physiopathological and clinical connections between MASLD and PAD: this dangerous link could be ameliorated by lifestyle changes and specific therapy for MASLD/PAD to reduce cardiovascular risk.

MASLD and PAD recognize, at least in part, overlapping etiopathogenetic bases (see Figure 2). Even from a clinical point of view, some comorbidities are frequently associated with both MASLD and PAD. Compared to other manifestations of atherosclerotic disease, PAD continues to be a little-known and little-researched entity both among various medical specialists and among patients. When a patient suffering from MASLD is identified, a vascular screening should be indicated, firstly through the ABI index, and secondly through an echo-color Doppler evaluation of the carotid, aortic and lower limb areas. Therefore, MASLD and PAD can coexist in the same patients, and the diagnostic–therapeutic effort must always be aimed at global patient care with the use of the latest imaging techniques and new drugs available, while waiting for future studies that can better clarify the relationship between MASLD and PAD and give us more targeted therapeutic indications.

## Figures and Tables

**Table 1:** Summary of principal studies linking MASLD and CV diseases.

Study (First Author, Year)	Population Size	MACE	HR/RR/OR (95% CI)
Mantovani et al. (2021) [2]	5,802,226 99,668 patients (with fatal and non-fatal CVE)	CVE	HR: 2.50 (1.68–3.72)
Koulaouzidis et al. (2021) [3]	10,060	CAC	OR: 1.5 (1.34–1.68)
Alon et al. (2022) [4]	>2.5 milion	ischemic stroke	OR: 1.41 (1.29–1.55)
		myocardial infarction	OR: 1.66 (1.39–1.99)
		heart failure	OR: 1.62 (1.43–1.84)
		atrial fibrillation	OR: 1.27 (1.18–1.37)
Lee et al. (2021) [5]	9,584,399		
	NAFLD group		HR: 1.09 (1.03–1.15)
	MAFLD group	myocardial infarction, ischemic stroke, heart failure, CV death	HR: 1.43 (1.41–1.45)
	Both conditions		HR: 1.56 (1.54–1.58)
Fu et al. (2023) [7]	>10 millions	CVE	HR: 1.49 (1.34–1.64)
		stroke	HR: 1.55 (1.37–1.73)
		carotid atherosclerosis	OR: 1.18 (1.00–1.38)
		PAD	OR: 1.32 (1.05–1.68)
		cardiovascular mortality	HR: 1.28 (1.03–1.53)
		total mortality	HR: 1.24 (1.13–1.34)
		cancer-related mortality	HR: 1.27 (1.01–1.54)
		liver-related mortality	HR: 2.76 (1.07–7.13)
		CKD	HR: 1.53 (1.38–1.68)
		HF	HR: 1.67 (1.58–1.76)
		obstructive sleep apnea	OR: 6.80 (1.81–25.6)
		all-cause mortality	HR: 1.32 (1.09–1.59)
		CV mortality	HR: 1.22 (1.06–1.41)
Bisaccia et al. (2023) [8]	10,592,851	all-cause mortality	OR: 1.14 (0.78–1.68)
		cardiovascular mortality	OR: 1.13 (0.57–2.23)
		myocardial infarction	OR: 1.6 (1.5–1.7)
		stroke	OR: 1.6 (1.2–2.1)
		atrial fibrillation	OR: 1.7 (1.2–2.3)
		major adverse CVE and cerebrovascular events	OR: 2.3 (1.3–4.2)
Jamalinia et al. (2023) [9]	4725	subclinical atherosclerosis	OR: 2.18 (1.62–2.93)
		fibrosis stage ≥ F1	OR: 1.64 (1.22–2.20)
		fibrosis stage ≥ F2	OR: 2.22 (1.37–3.62)
		fibrosis stage ≥ F3	OR: 3.42 (1.81–6.46)

**Table 2:** Clinical overview and characteristics of PAD.

PAD Clinical Overview	
Symptoms	- Intermittent claudication - Declining walking ability - Weakness and leg discomfort
Risk Factors	- Diabetes - Smoking habits - Hypertension - Dyslipidemia - Sedentary lifestyle
Link with Blood Pressure	- Systolic BP has a graded association with PAD - Diastolic BP increases risk only above 90 mmHg
Inflammatory Markers	- IL-6 and CRP could be elevated in symptomatic PAD patients
Link with MASLD and Metabolic Syndrome	- MASLD is linked to increased PAD risk and atherosclerosis - Insulin resistance contributes to both PAD and fibrosis progression
ABI/TBI Testing	- ABI measures systolic pressure in arms/legs; ≤0.90 indicates PAD - TBI is used when ABI > 1.40 due to arterial stiffness
Screening Recommendations	- ABI testing recommended for adults over 65 or over 50 with risk factors - Comprehensive screening can reduce mortality
Diagnostic Methods	- ABI is non-invasive, sensitive, and specific for diagnosing PAD - TBI is diagnostic for PAD when ≤0.70 - Vascular US is best diagnostic non-invasive method - Advanced imaging (CT, MRI) assists in surgical decisions
Socio-economic Factors	- Low education, income and isolation increase PAD risk regardless of other factors

**Table 3:** Summary of clinical evidence between MASLD and PAD.

Study (First Author, Year)	Population Size	Prevalence of PAD/MACCEs	(%)	HR/RR/OR (95% CI)	*p* Value
Song et al. (2023) [37]	102,115	MAFLD group	2.70%	OR: 1.30 (1.19–1.42)	*p* < 0.001
		non MAFLD group	2.20%		
Subgroup of prospective cohort	6833			HR: 1.67 (1.17–2.38)	*p* = 0.005
Huang et al. (2024) [70]	889	DFUs patients with MAFLD non-MAFLD			
		MAFLD group		2.64-fold increased risk for MACCEs HR: 3.64 (2.358–5.62)	*p* = 0.001
